# Recent advances in primary immunodeficiency: from molecular diagnosis to treatment

**DOI:** 10.12688/f1000research.21553.1

**Published:** 2020-03-19

**Authors:** Giorgia Bucciol, Isabelle Meyts

**Affiliations:** 1 Inborn Errors of Immunity, Department of Immunology, Microbiology and Transplantation, KU Leuven, Herestraat 49, Leuven, 3000, Belgium; 2 Childhood Immunology, Department of Pediatrics, University Hospitals Leuven, ERN-RITA Core Member, Herestraat 49, Leuven, 3000, Belgium

**Keywords:** primary immunodeficiency, inborn error of immunity, next generation sequencing, targeted therapy, hematopoietic stem cell transplantation

## Abstract

The technological advances in diagnostics and therapy of primary immunodeficiency are progressing at a fast pace. This review examines recent developments in the field of inborn errors of immunity, from their definition to their treatment. We will summarize the challenges posed by the growth of next-generation sequencing in the clinical setting, touch briefly on the expansion of the concept of inborn errors of immunity beyond the classic immune system realm, and finally review current developments in targeted therapies, stem cell transplantation, and gene therapy.

## Introduction

Perhaps more than other medical disciplines, the field of primary immunodeficiency is expanding rapidly thanks to recent advances in sequencing, gene editing tools, and the introduction of new biological drugs and small molecules that target specific checkpoints relevant to immunity, inflammation, and cancer. We have witnessed, first of all, a rapid rise in the number of described monogenic inborn errors of immunity (IEIs), and more than 400 distinct defects were included in the International Union of Immunological Societies (IUIS) classification of 2019 ^1^. This represents an increase of 200 new genes and diseases in 10 years and reveals a paradigm shift from “primary immunodeficiencies” as fundamental defects in the immune response to infection toward the broader concept of “inborn errors of immunity” as a comprehensive group of different phenotypes, including infection, autoinflammation, autoimmunity, allergy, and malignancy ^1, 2^. Indeed, a relatively new concept in IEIs is immune dysregulation caused by the innate components of immunity, in juxtaposition with adaptive immunity-driven autoimmunity. Autoinflammatory disorders are caused by an over-activation of pro-inflammatory cytokines or pathways, mostly components of the inflammasomes. They typically manifest as fevers, skin rashes, systemic inflammation, and variable arthritis and lymphadenopathy, although some forms can also present with immunodeficiency and other clinical phenotypes ^3^. A subgroup of these disorders is represented by interferonopathies, characterized by constitutively activated type I interferon (IFN) responses ^4^.

New insight into the pathogenesis of IEIs has introduced targeted treatments next to substitution and symptomatic therapy (immunoglobulin replacement, antimicrobial and anti-inflammatory, or immunosuppressive treatments) on one side and replacement of the flawed immune system by hematopoietic stem cell transplantation (HSCT) or gene therapy on the other ^2^. Moreover, the latter have greatly benefited from the new advances in cell and gene manipulation, expanding the number of diseases to be successfully treated and increasing the survival chances of affected individuals ^5^. In this review, we focus on some of the most recent advances over the last 3 years in the diagnosis and therapy of human IEIs.

## Molecular diagnosis of inborn errors of immunity

Sequencing in general and next-generation sequencing (NGS) techniques in particular are becoming technically more accurate, fast, affordable and therefore widely available to researchers and physicians. Apart from the obvious result of increasing the sheer number of defined IEIs, this rapid expansion has highlighted the phenotypic heterogeneity and genetic pleiotropy of these disorders. On the other hand, many novel IEIs are studied in a single kindred or a small number of kindreds, thus providing little information on the penetrance and phenotype ^1^. Moreover, the pathophysiology often remains to be unraveled. For example, heterozygous mutations in cell division cycle 42 ( *CDC42*), encoding a small GTP/GDP-binding protein involved in eukaryotic actin cytoskeleton dynamics, cause a wide range of different developmental phenotypes with or without autoinflammation, depending on the affected protein domain ^6– 12^. Patients with biallelic mutations in lariat debranching enzyme 1 ( *DBR1*) have a very rare brainstem viral encephalitis through a defect of cell-intrinsic immunity that is still entirely unexplained ^13^. A relevant example of phenotypic heterogeneity is the deficiency of adenosine deaminase 2 (DADA2): originally reported as a small-vessel vasculitis manifesting with polyarteritis nodosa, livedo racemosa, stroke, and mild immunodeficiency, the phenotype has significantly expanded to include pure red cell aplasia, other cytopenias, lymphoproliferation, and lymphoma ^14– 30^.

In at least 10 genes responsible for IEI, both gain-of-function (GOF) and loss-of-function (LOF) mutations have been described, resulting in different biological effects and clinical phenotypes ( Table 1) ^31– 55^.

**Table 1.** PID genes in which GOF and LOF mutations have been described.

Protein	Gene	OMIM n. disease	Inheritance	Immunological phenotype	Infectious phenotype	Non-infectious phenotype	Autoinflammation	Therapy
STAT1	*STAT1*	613796	AR Complete/partial LOF	Deficient intracellular pathogen killing by monocytes and T cells; impaired IFN type I response to viral infections	Lethal viral diseases and susceptibility to mycobacterial infection	N/A	No	N/A
		614892	AD Dominant-negative LOF	Deficient intracellular pathogen killing by monocytes and T cells	Mendelian susceptibility to mycobacterial disease	N/A	No	IFN-γ
		614162	AD GOF	Low Th17 proportions ± low memory B cells	Chronic mucocutaneous candidiasis, bacterial and viral infections, invasive fungal infections, mycobacterial disease	Intracranial aneurysms, autoimmunity, enteropathy, bronchiectasis	Yes	JAK inhibitor (ruxolitinib) HSCT
CARD11	*CARD11*	615206	AR LOF	Defective NF-κB signaling with B- and T-cell functional impairment, hypogammaglobulinemia	*Pneumocystis jirovecii* pneumonia	N/A	No	HSCT
		617638	AD Dominant-negative LOF	Defects in T-cell activation, increased IgE, and eosinophilia	Recurrent or severe infections, including pneumonia, molluscum, abscesses, and bacteremia	Moderate to severe atopic dermatitis, asthma, allergies, ulcerative colitis, T-cell lymphoma	No	N/A
		616452	AD GOF	B-cell expansion with NF-κB and T-cell anergy (BENTA), decreased T-cell proliferative responses and vaccine responses	Recurrent upper respiratory infections	Splenomegaly, lymphadenopathy, autoimmunity, chronic lymphocytic leukemia	No	N/A
WASP	*WAS*	301000	X-linked recessive LOF	Lymphopenia, hypogammaglobulinemia, eosinophilia, decreased vaccine responses	Recurrent bacterial infections, molluscum	Microthrombocytopenia, eczema, autoimmunity, hematological malignancies, inflammatory bowel disease	Yes	HSCT, gene therapy
		300299	X-linked recessive GOF	Severe congenital neutropenia, monocytopenia, natural killer cell deficiency, increased CD8 ^+^ T cells	Severe bacterial infections	N/A	No	Granulocyte- colony- stimulating factor
MDA5	*IFIH1*	-	AR/AD LOF	Respiratory epithelial cells and fibroblasts	Rhinovirus and other respiratory viral infections	N/A	No	N/A
		615846	GOF	Aicardi–Goutieres syndrome: type I interferonopathy	Not reported	Pseudo-TORCH syndrome: basal ganglia calcification, microcephaly, developmental delay, spasticity, autoimmunity	Yes	JAK inhibitor (ruxolitinib)
STAT3	*STAT3*	147060	AD Dominant-negative LOF	Hyper IgE syndrome, eosinophilia, T-cell defect with Th17 deficiency, decreased vaccine responses	Recurrent Staphylococcal infections, fungal infections,	Coarse facial appearance, abnormal dentition, hyperextensibility of the joints, and bone fractures	No	HSCT?
		615952	AD GOF	T-cell lymphopenia, hypogammaglobulinemia, increased double-negative T cells	Recurrent infections, including fungal infections	Autoimmunity, dermatitis, arthritis, interstitial lung disease, short stature, lymphadenopathy, splenomegaly	Yes	HSCT?
STAT2	*STAT2*	616636	AR LOF	Defective type I IFN-STAT signaling	Increased susceptibility to viral infections and to disseminated disease after live vaccines (measles)	Defect of mitochondrial fission and fusion	No	N/A
		-	AR GOF	Type I interferonopathy	Not reported	Severe early-onset autoinflammation	Yes	N/A (possibly JAK inhibitor)
Complement factor B	*CFB*	615561	AR LOF	Inactive alternative complement pathway	Infections with encapsulated bacteria	N/A	No	Vaccination, prophylactic antibiotics
		612924	AD GOF	Decreased C3 and increased C3b	Not reported	Atypical hemolytic uremic syndrome	No	N/A
C3	*C3*	613779	AR LOF	C3 deficiency	Recurrent bacterial infections, mainly by Gram-negative bacteria	Immune complex-mediated autoimmune disease, glomerulonephritis	No	N/A
		612925	AD GOF/LOF	None	Not reported	Atypical hemolytic uremic syndrome	No	N/A
JAK1	*JAK1*	-	AR LOF	Progressive T-cell lymphopenia, increased IgG	Mendelian susceptibility to mycobacterial disease, warts, parasitic and fungal infections	Early-onset bladder carcinoma	No	N/A
		-	AD GOF	Eosinophilia	Not reported	Hepatosplenomegaly, liver cysts, severe atopic dermatitis, allergy, autoimmunity, failure to thrive	No	JAK inhibitor (ruxolitinib)
ZAP70	*ZAP70*	269840	AR LOF	Selective T-cell defect with CD8 ^+^ deficiency and CD4 ^+^ impairment	Severe combined immunodeficiency phenotype: bacterial, viral and opportunistic infections	N/A	No	HSCT
		617006	AR LOF + GOF	Mild T- and B-cell lymphopenia, reduced CD8 ^+^ T cells, decreased T-cell proliferative responses	Not reported	Bullous pemphigoid, inflammatory bowel disease, autoimmunity	Yes	HSCT

AD, autosomal dominant; AR, autosomal recessive; GOF, gain of function; HSCT, hematopoietic stem cell transplantation; IFN, interferon; JAK, Janus kinase; LOF, loss of function; N/A, not available/not applicable; NF-κB, nuclear factor kappa B; STAT, signal transducer and activator of transcription; Th, T helper; TORCH, toxoplasmosis, rubella, cytomegalovirus, herpes.

Mutations of signal transducer and activator of transcription 1 ( *STAT1*) are exemplary. Biallelic LOF mutations cause either complete or partial STAT1 deficiency: the first impairs type I and II IFN responses and produces a severe phenotype of mycobacterial and viral susceptibility, which invariably is fatal if not corrected by HSCT; the second has a similar but milder presentation ^46, 47, 56– 60^. Heterozygous mutations with a dominant-negative LOF effect impair mainly type II IFN signaling and cause mendelian susceptibility to mycobacterial disease (MSMD) and salmonellosis ^48, 57, 61^. Finally, heterozygous *STAT1* GOF mutations affect interleukin-17 (IL-17) immunity and cause autosomal dominant (AD) chronic mucocutaneous candidiasis (CMC), bacterial and viral infections, autoimmunity, and cerebral aneurysms ^49, 50, 57, 62^. A similar situation can be seen in defects of caspase recruitment domain family 11 ( *CARD11*). Homozygous null mutations in fact cause a profound combined immunodeficiency, heterozygous dominant-negative LOF mutations cause combined immunodeficiency with severe atopic disease, and heterozygous GOF mutations instead cause B-cell expansion with nuclear factor kappa B (NF-κB) and T-cell anergy (BENTA) disease ^51– 55, 63, 64^. These examples of additional phenotypes progressively connected to different mutations in the same genes highlight the risk of labeling heterozygous variants as irrelevant to the observed clinical phenotype without functional testing.

With NGS methods routinely available for diagnosis and with a shift from the fixed gene panel and whole exome sequencing (WES) toward whole genome sequencing (WGS), we also learned that deep intronic variants can be disease-causing ^65, 66^. For example, various deep intronic mutations in *UNC13D*, underlying familial hemophagocytic lymphohistiocytosis (HLH), are very commonly found in patients of European, North American, or Asian origin ^67– 71^. Similarly, intronic mutations were found in a number of IEI-causing genes: in *IL7R* and Janus kinase 3 ( *JAK3*) in patients with severe combined immunodeficiency (SCID); in *ZAP70*, encoding the zeta chain-associated protein kinase, in a child with severe T-cell immunodeficiency; in *STAT3* in a patient with hyper IgE syndrome; in the NF-κB essential modulator ( *NEMO*) in two patients with ectodermal dysplasia and immunodeficiency (EDA-ID); in *POLA1*, encoding DNA polymerase α, in patients with X-linked recessive reticulate pigmentary disorder (XLPDR); in *CYBB*, encoding p91-PHOX, in patients with chronic granulomatous disease (CGD); in *ATM* in patients with ataxia-telangiectasia; and in *CD40LG* in patients with hyper IgM syndrome ^72– 85^. RNA sequencing can be an invaluable tool in the validation of these deep intronic variants but also of synonymous and splice-site variants ^75, 76, 86, 87^. In particular, it can highlight the partial or complete loss of gene expression in the proband compared with controls, which would be missed by traditional sequencing. The increase in diagnostic rate of rare diseases obtained by WGS versus WES can be as high as 6 to 11%, and the use of tissue-specific RNA sequencing can aid the diagnostic process and help clarify the pathophysiology of the disease ^86, 87^. Interestingly, in the case of monogenic rare diseases, RNA sequencing as the first molecular approach led to a diagnosis in 7 to 17% of cases ^87^. No hematological/immunological disorders were among those successfully diagnosed in this cohort, which consisted mostly of patients affected by neuromuscular disorders. A lower success rate in IEIs could also be a reflection of the difficulty of comparing normal controls with patients with significant blood cell abnormalities.

The benefit of diagnostic NGS comes at a cost: the necessity of unequivocal functional validation of new variants, albeit in known IEI-related genes. Still too often the pathogenicity of a given variant is based only on the available *in silico* prediction tools, which can be misleading. Indeed, the validation of variants of unknown significance represents one of the biggest hurdles in making WES and NGS technologies the norm in clinical practice. This is because of not only the financial cost but also the time and competence required first to analyze and study a variant and afterwards to interpret the results and translate them into clinical treatment and care. More than anything, this calls for collaboration and sharing of data for the benefit of the patients. With limited research resources, the further development of more robust prediction tools/validation models becomes a necessity. Finally, as physician-scientists, we wish to stress the importance of the clinical phenotype as a beacon in the era of so-called unbiased sequencing approaches.

## Inborn errors of immunity in other diseases

As previously mentioned, the concept of what constitutes an immunodeficiency is steadily shifting from a focus on defects of hematopoietic immune system components, such as leukocytes and immunoglobulins, to conditions affecting immunity at the organism level ^88^. Classic examples are cystic fibrosis and sickle cell disease: the first is a chloride channel defect with primarily pulmonary and digestive manifestations, the second is a red blood cell disease caused by mutations in the β-globin gene, and both have recurrent and life-threatening infections as major symptoms (secondary to functional asplenia in the case of sickle cell disease) ^88– 93^. All cells and tissues exert essential host defense functions that range from physical and chemical barriers to pathogen sensing, cytokine production, and protein secretion and activation of the immune response upon infection, representing cell-intrinsic immunity. Infection and inflammation in fact can arise in several disorders of organs other than the classic hematopoietic immune system. The importance of these cell-specific immune responses is apparent when we consider those IEI characterized by the involvement of a single non-hematopoietic tissue, such as the central nervous system (CNS)-specific lack of resistance against herpesviruses in case of mutations of the Toll-like receptor 3/IFN pathway, or the keratinocyte-restricted susceptibility to beta-papillomavirus infections in epidermodysplasia verruciformis (due to null mutations in transmembrane channel-like protein 6 [ *TMC6*], *TMC8* [encoding EVER1 and EVER2, respectively], or *CIB1*, encoding calcium- and integrin-binding protein 1) ^94– 104^. More generally, inherited defects of the skin or mucosal barriers cause at the very least a detrimental secondary effect on immune protection, as well exemplified by genodermatoses and inflammatory bowel diseases.

Several defects of the skin barrier frequently cause secondary infection of the affected epithelia and can also present with additional features resembling known IEIs ^105^, such as Netherton syndrome (caused by autosomal recessive [AR] defects in *SPINK5*), hyper IgE syndrome (caused by defects in *STAT3*, *DOCK8*, *IL6ST*, *ZNF341*, or phosphoglucomutase 3 [ *PGM3*]), or immunodysregulation polyendocrinopathy enteropathy X-linked (IPEX) syndrome (caused by X-linked *FOXP3* defects). Defects in desmoglein 1 (DSG1) or desmoplakin (DSP), structural desmosomal proteins, cause severe dermatitis, multiple allergies, and metabolic wasting (SAM) syndrome, a severe multisystemic disease resembling IPEX syndrome at least clinically ^106, 107^. Recent reports have highlighted the complex clinical and immunological phenotype of this disease, including recurrent sepsis and mucocutaneous HSV-1 infection in a patient and T helper 1 (Th1)/Th17/IL-23 skewing in the skin and Th17/IL-22 skewing in the blood of another patient, which merit further research ^108– 110^.

The immunological effects of glycosylation defects and metabolic diseases have also recently gained interest in the IEI field. Glycosylation is crucial in many mechanisms of the immune response, such as pathogen recognition and cell–cell interaction, and glycoimmunology is a rapidly expanding field of research ^111– 119^. Among the currently known congenital disorders of glycosylation (CDGs) (around 133 heterogenous diseases), 23 show a relevant degree of immune impairment; in 10 of these, immunodeficiency is a prominent trait of the disease ^118^. Two of these CDGs manifest as severe congenital neutropenia caused by defects in Jagunal homolog 1 ( *JAGN1*) and glucose-6-phosphatase catalytic 3 ( *G6PC3*), one is a leukocyte adhesion deficiency type II due to defects in solute carrier family 35 member C1 ( *SLC35C1*), one is a glycogen storage disease type I with neutropenia or neutrophil dysfunction (or both) caused by defects in *SLC37A4*, and the others show various degrees of lymphocyte and immunoglobulin impairment ^118, 120– 125^. Additionally, AR *PGM3* mutations cause glycosylation defects that lead to atopy, immune deficiency with hyper IgE, autoimmunity, and neurocognitive impairment ^126^. Finally, a recent study highlighted the role of impaired glycosylation in the pathogenesis of X-linked immunodeficiency with magnesium defect, Epstein–Barr virus (EBV) infection, and neoplasia (XMEN) disease, caused by hemizygous LOF mutations in the magnesium transporter 1 ( *MAGT1*) gene ^127, 128^. On the other side, the study of differentially glycosylated antibodies and their diverse antigen reactivity will teach us more about the fine tuning of the immune responses by glycosylation ^129^.

## Targeted therapies for inborn errors of immunity

Recent developments in molecular studies have allowed the identification of several possible targets for specific therapeutic interventions. Targeted therapies comprise monoclonal antibodies (mAbs) and small molecules, such as cytokines or cytokine inhibitors, employed to up- or down-regulate a particular pathway, depending on the need. They can be used instead of or in combination with traditional immunosuppressant/immunomodulating agents, also as a bridge to definitive treatment, such as HSCT or gene therapy. Well-known targeted therapies are rituximab (anti-CD20) to treat autoimmune and lymphoproliferative manifestations; enzyme replacement therapy with pegylated bovine adenosine deaminase (ADA) to treat ADA-SCID; IFN-γ to increase superoxide production in the monocytes/macrophages of patients with CGD and to enhance anti-mycobacterial immunity in patients with defects of the IFN-γ/IL-12R pathway; anti-tumor necrosis factor alpha (anti-TNFα) to treat defects of the immunoproteasome causing an autoinflammatory syndrome; IL-1 signal antagonists to treat inflammasome disorders; and finally mechanistic target of rapamycin (mTOR) inhibitors to control aberrant proliferation of effector T cells in various immune dysregulation disorders, such as in IPEX, or to downregulate increased mTOR signaling, such as in activated phosphoinositide 3-kinase δ (PI3Kδ) syndrome (APDS, or PASLI) ^130– 136^.

The latest molecular defects to be targeted in the context of IEI therapy are *PI3Kδ* activating mutations, cytotoxic T lymphocyte-associated antigen 4 ( *CTLA4*) haploinsufficiency and the closely related lipopolysaccharide (LPS)-responsive beige-like anchor ( *LRBA*) deficiency, *STAT1* GOF mutations, *STAT3* GOF mutations, IFN-γ activation in the context of HLH, and activating defects in NLR family CARD domain-containing 4 ( *NCLR4*).

### Selective inhibitors of PI3Kδ

Patients with APDS present with recurrent respiratory tract infections, herpesvirus infections, lymphoproliferation, autoimmunity and a characteristic immunophenotype with impaired class-switch recombination, hypogammaglobulinemia, T-cell hyperactivation, and senescence with loss of naïve T cells and predominance of CD8 ^+^ cells ^136– 142^. Two selective PI3Kδ inhibitors have been tested on patients with APDS: Leniolisib, a potent oral inhibitor of the p110δ subunit of PI3Kδ (ClinicalTrials.gov Identifier: NCT02435173), has shown significant effects on general well-being, lymphoproliferation, and immunological markers, such as normalization of B-cell subsets, reduction of senescent T cells, and reduction of IgM and of inflammatory cytokines ^143, 144^. Nemiralisib, an inhaled PI3Kδ inhibitor (ClinicalTrials.gov Identifier: NCT02593539), could benefit patients primarily affected by airway infection and bronchiectasis and is being trialed in patients with APDS ^145^.

### CTLA4-IgG fusion proteins

CTLA4 haploinsufficiency causes impaired T-cell suppressor function, CD4 ^+^ T-cell deficiency, progressive loss of B cells, and increase in autoreactive B cells. Clinical manifestations are recurrent sinopulmonary and viral infections, severe autoimmunity with cytopenia, lymphocytic interstitial lung disease, enteropathy, colitis, and lymphoproliferation with lymphocytic infiltration of solid organs, such as the brain and endocrine glands ^146– 148^. Although the pathophysiology of LRBA deficiency is still incompletely elucidated, LRBA acts a chaperone protein for CTLA4 to allow its recycling in endosomes. LRBA deficiency causes increased degradation of CTLA4 and a combined immunodeficiency disorder with hypogammaglobulinemia, infections, and severe autoimmune features, including cytopenias, enteropathy, lymphoproliferation, hepatitis, diabetes, polyarthritis, and alopecia ^149– 151^. CTLA4-IgG fusion proteins abatacept and belatacept have demonstrated significant results in restoring the impaired checkpoint balance and reducing symptoms in CTLA4- and LRBA-deficient patients. In particular, they could restore regulatory T (Treg) cell function and halt or (partially) resolve autoimmune, lymphoproliferative, and inflammatory symptoms, including interstitial lung disease, enteropathy, cytopenias, and other autoimmune manifestations ^148, 152– 154^.

### JAK/STAT inhibitors (Jakinibs)

GOF mutations in *STAT1* cause CMC, bacterial and viral infections, autoimmunity, immune dysregulation, and a higher risk of cerebral aneurysms and vasculopathy ^49, 50, 62^. STAT1 is a signal transducer downstream from type I and II IFN receptors and other cytokine receptors, such as IL-21R and IL-2R. GOF defects in *STAT3* cause a severe autoimmune syndrome with growth impairment, lymphoproliferation, and inflammatory features ^33, 155, 156^. STAT3 also signals downstream from type I, II, and III IFN receptors, IL-10R, IL-23R, and IL-6R. These receptors activate STAT1 and STAT3 through JAK proteins (JAK1, JAK2, and JAK3), thus initiating the transcription of several factors relevant to the immune response, cell proliferation, differentiation, and survival. Jakinibs are small molecules that inhibit the signal transduction through JAK proteins: tofacitinib preferentially inhibits JAK1 and JAK3, ruxolitinib and baricitinib inhibit JAK1 and JAK2, and many other Jakinibs with different JAK specificity were recently discovered ^157, 158^. Ruxolitinib and tofacitinib have been used in patients with STAT1 or STAT3 GOF mutations, mostly with significant clinical improvement on fungal infections, autoimmune manifestations, and lymphoproliferation ^159– 164^. Selective inhibition of IL-6R with tocilizumab is also a valid targeted therapy in STAT3 GOF defects, especially when combined with Jakinibs ^155, 159^.

### Anti-IFN-γ mAb

HLH is a disease characterized by hyperinflammation and immune dysregulation and is fatal if not treated. It can be primary, owing to defects of cytotoxic T cells, natural killer (NK) cells, or genes required for EBV control and clearance, or secondary, owing to an exaggerated response to viral infections, malignancies, or rheumatologic disorders. Its manifestations are fever, systemic inflammation, splenomegaly, cytopenias, and hemophagocytosis in the bone marrow, with or without CNS involvement. Isolated CNS forms have also been described ^165^. Classic treatment targets the life-threatening inflammation and includes steroid therapy, chemotherapy (etoposide and intrathecal methotrexate), and HSCT ^166, 167^. Anti-CD52 mAb alemtuzumab has been proven effective as salvage therapy of refractory HLH ^168^. Emapalumab is a recently developed mAb against IFN-γ and has been tested for treatment of HLH. An international phase II/III trial (ClinicalTrials.gov Identifier: NCT01818492) showed a good safety profile and efficacy, an overall response rate of 65%, and a complete response in 26% of cases ^169, 170^. However, caution is warranted as the number of patients who have received treatment is still very small and the concomitant therapies were significant.

### Future applications

Treatment with Jakinibs could benefit other disorders characterized by inflammation and hyperactivation of the IFN pathways, such as interferonopathies, stimulator of interferon genes (STING)-associated vasculopathy with onset in infancy, and HLH ^171– 173^. Ruxolitinib was recently used in two patients with HLH: both showed a decrease of inflammatory markers, but only one recovered from cytopenias and survived long enough to receive HSCT ^174, 175^. A phase I trial evaluating ruxolitinib for patients with HLH is now recruiting (ClinicalTrials.gov Identifier: NCT02400463).

## Recent advances in hematopoietic stem cell transplantation and gene therapy

Since they are largely due to intrinsic defects of hematopoietic cells or their descendant, the mature blood cells, HSCT represents the treatment of choice for many IEIs. Thanks to better and faster molecular diagnosis, improvement in conditioning practices, better donor selection and graft manipulation, and development of more successful supportive therapies to guarantee engraftment while fending off infections and graft-versus-host disease (GvHD), the overall survival of immunodeficient patients after HSCT has steadily improved up to over 80% ^176– 179^. What then are the challenges and future perspectives in this field?

### Newborn screening and hematopoietic stem cell transplantation

Newborn screening has changed the landscape of HSCT for SCID because early diagnosis allows early transplantation with a smaller burden of infection. Based on a T-cell receptor excision circle assay performed on the dried blood spots taken after birth for the other newborn screenings, it has been extensively (though not uniformly) adopted in Europe and the US in the last decade ^180, 181^. It allows the identification of T-cell development disorders that affect the diversity of T-cell receptor recombination, namely SCIDs. Although there is no consensus about the influence of the diagnostic method on overall survival, HSCT before the age of 3.5 months is associated with fewer infectious complications and higher survival, and event-free survival is better for children identified by newborn screening if uninfected at the time of transplant ^182– 184^. Indeed, the current challenge lies in protecting SCID babies identified by newborn screening from infection prior to transplantation. Early diagnosis of SCID also poses therapeutic dilemmas: while owing to the high risk of infections delaying HSCT is not recommended, there are no data on long-term neurotoxicity of conditioning regimens in young infants. Moreover, the genetic diagnosis may not be available in time to adapt the transplantation plan accordingly, and patients with radiosensitive SCIDs such as Artemis or DNA ligase IV deficiency could experience severe early toxicity and long-term sequelae after standard conditioning since chemotherapy causes DNA breakages in non-hematopoietic cells and tissues.

### Conditioning

Although the level of chimerism needed to correct IEIs is not precisely defined, most IEIs do not require full donor chimerism, explaining why modified myeloablative and reduced intensity conditioning has become the practice of choice in many centers to reduce acute and long-term toxicity ^185^. A promising line of research is represented by targeted antibody-based conditioning strategies, which are probably going to replace chemotherapy-based regimens in the near future ^186^. These therapeutic agents work by selectively targeting bone marrow cells and leukocytes for apoptosis, either by disrupting the physiological cell proliferation/cell death cycle or by delivering a radioisotope or a drug toxic to the cell. CD45 (or common leukocyte antigen) is selectively expressed on hematopoietic cells. Rat anti-CD45 mAbs were used together with anti-CD52 (alemtuzumab) and cyclophosphamide in a small group of patients with IEIs and pre-existing organ toxicity with good outcome ^187^. Further trials of radioisotope-labeled anti-CD45 mAb are ongoing in patients with myeloid disorders (ClinicalTrials.gov Identifiers: NCT00119366 and NCT01300572), and antibody–drug conjugates have shown promising results in several animal models ^188– 192^. Similarly, a mAb against CD117 (or c-Kit), necessary for survival, proliferation, and differentiation of hematopoietic stem cells and early progenitors, has demonstrated good preclinical results in animal models and is being tested in patients with SCID (ClinicalTrials.gov Identifier: NCT02963064) ^193– 195^.

### Gene therapy

Hematopoietic stem cells are the perfect candidate for genetic manipulation. Indeed, gene therapy has been applied to the treatment of blood-specific disorders, including IEIs, for at least three decades ^196^. The first attempts at gene therapy in the field of IEIs began in the ’90s and targeted ADA-SCID and X-SCID, caused by defects in the common gamma chain of IL-2 receptor, and subsequently chronic granulomatous disease and Wiskott–Aldrich syndrome ^196– 202^. The technique is based on autologous stem cell infusion after *in vitro* correction of the molecular defect by gene addition, eliminating the risk of GvHD and making it an appealing alternative to HSCT. The original trials were successful in terms of genetic defect correction and clinical benefit, but the gamma-retroviral vectors in use were associated with leukemia or monoclonal expansion, except in the case of ADA-SCID ^196, 197, 203– 206^. These serious adverse events seem to have been overcome by the introduction of safer self-inactivating lentiviral vectors, and new studies are under way for a number of IEIs, including RAG-SCID, X-linked lymphoproliferative syndrome, and perforin deficiency ^197^. Whereas the earlier trials were based on gene addition (that is, the introduction by means of a viral vector of the wild-type gene in the host genome), recent approaches have investigated gene editing as a way to correct a molecular defect *in situ*. CRISPR/Cas9 has become a particularly trending method, especially if paired with the delivery of a donor template (the wild-type cDNA of the gene of interest) via a viral vector that integrates after the natural promotor of the gene to maintain physiological expression and regulation. At the moment, transfection efficiency is still lower with gene editing methods than with gene addition via lentiviral transduction, mainly due to high cell mortality during transfection procedures, and on-target and off-target side effects must be better understood ^196, 197^. This technique is in a preclinical stage or phase I/II trials in several IEIs, including X-linked SCID, hyper IgM syndrome, CGD, and X-linked agammaglobulinemia, and looks very promising in the context of GOF mutations ^196, 197, 207– 211^. Moreover, so-called T-cell gene therapy is being studied for the correction of T cell–intrinsic defects with encouraging results, and trials are under way for IPEX, hyper IgM syndrome, X-linked lymphoproliferative disease, Munc 13-4 deficiency, and perforin deficiency ^207, 212– 216^. Still at a preclinical phase is base editing, a new development in gene editing techniques that would allow the correction of single point mutations without requiring the DNA cleaving step ^217, 218^. Future studies will need to clarify what the best positioning of gene therapy approaches versus HSCT versus conservative therapy is.

## Conclusions

In this review, we have tried to provide a timely overview of recent advances in the diagnosis and treatment of primary immunodeficiencies/IEIs. With rapidly evolving molecular techniques as the leading force, the end of progress is not yet in sight. Indeed, it can be expected that we are on the verge of a further breakthrough of knowledge in primary immunodeficiency.

Challenges will lie in making these treatments and diagnostics tools available to as many patients as possible and to tailor them to specific needs. Undoubtedly, primary immunodeficiencies/IEIs as experiments of nature will continue to teach us about the magnificent and still-underestimated complexity of the human immune system.

## Abbreviations

AD, autosomal dominant; ADA, adenosine deaminase; APDS, activated phosphoinositide 3-kinase δ syndrome; AR, autosomal recessive; CARD11, caspase recruitment domain family 11; CDG, congenital disorder of glycosylation; CGD, chronic granulomatous disease; CMC, chronic mucocutaneous candidiasis; CNS, central nervous system; CTLA4, cytotoxic T lymphocyte-associated antigen 4; DOCK8, dedicator of cytokinesis 8; EBV, Epstein–Barr virus; FOXP3, forkhead box P3; GOF, gain of function; GvHD, graft-versus-host disease; HLH, hemophagocytic lymphohistiocytosis; HSCT, hematopoietic stem cell transplantation; IEI, inborn error of immunity; IFN, interferon; IL, interleukin; IL6ST, interleukin 6 signal transducer; IPEX, immunodysregulation polyendocrinopathy enteropathy X-linked; JAK, Janus kinase; LOF, loss of function; LRBA, lipopolysaccharide-responsive beige-like anchor; mAb, monoclonal antibody; mTOR, mechanistic target of rapamycin; NF-κB, nuclear factor kappa B; NGS, next-generation sequencing; PGM3, phosphoglucomutase 3; PI3Kδ, phosphoinositide 3-kinase δ; SCID, severe combined immunodeficiency; SLC37A4, solute carrier family 37 member A4; SPINK5, serine protease inhibitor Kazal type 5; STAT, signal transducer and activator of transcription; Th, T helper; TMC, transmembrane channel-like protein; WES, whole exome sequencing; WGS, whole genome sequencing; ZNF341, zinc finger 341
